# Recent advances in M13 bacteriophage-based optical sensing applications

**DOI:** 10.1186/s40580-016-0087-5

**Published:** 2016-10-25

**Authors:** Inhong Kim, Jong-Sik Moon, Jin-Woo Oh

**Affiliations:** 1 Research Center for Energy Convergence Technology, Pusan National University, Busan, 46241 Republic of Korea; 2 BK21 Plus Division of Nano Convergence Technology, Pusan National University, Busan, 46241 Republic of Korea; 3 Department of Nanoenergy Engineering, Pusan National University, Busan, 46241 Republic of Korea

**Keywords:** M13 bacteriophage, Phage-based sensor, Immunofluorescence assay, SPR, FRET, SERS

## Abstract

Recently, M13 bacteriophage has started to be widely used as a functional nanomaterial for various electrical, chemical, or optical applications, such as battery components, photovoltaic cells, sensors, and optics. In addition, the use of M13 bacteriophage has expanded into novel research, such as exciton transporting. In these applications, the versatility of M13 phage is a result of its nontoxic, self-assembling, and specific binding properties. For these reasons, M13 phage is the most powerful candidate as a receptor for transducing chemical or optical phenomena of various analytes into electrical or optical signal. In this review, we will overview the recent progress in optical sensing applications of M13 phage. The structural and functional characters of M13 phage will be described and the recent results in optical sensing application using fluorescence, surface plasmon resonance, Förster resonance energy transfer, and surface enhanced Raman scattering will be outlined.

## Introduction

In general, a bio-chemical or a bio-optical sensor is a device to detect chemical or optical change in its biological medium. When the chemical or physical phenomena are measured, the quantitative information for the chemical and/or physical state within medium are provided by converting that into electrical or optical signal. Thus, a bio-chemical or a bio-optical sensor is a kind of a transducer using biomaterials that is used as a receptor for detection or measurement. Recently, bio-chemical or -optical sensors monitoring various substance or chemical constituent that is of interest such as nitro compound, peptide, nucleic acid, polymer, toxin, neurochemicals have been developed [1–15]. Alongside with these advances in biosensor applications, receptor materials that make it possible to transduce biological phenomena have also been extensively investigated in many fields. In these sensors, receptors are either integrated within or closely associated with a transducer interface providing the corresponding output [1]. In addition, transducers normally do not have specificity against a target analyte [16]. For that reason, the development of a sensor that is able to selectively detect target analytes is required [1]. Moreover, target-specificity is also an essential factor in the novel sensor development.

Recently, due to its nontoxic, self-assembling and specific binding properties, bacteriophage has been proven to be useful for the detection of target analytes in biomaterials by bio-chemical or bio-optical sensing applications. In addition, surface properties of bacteriophage, which is suitable as a receptor for the development of target-specific bio-sensor, can be controlled through genetic engineering. Employing this advantage, M13 bacteriophage (M13 phage) has expanded its use into novel research area, such as electrode, solar cell, environmental monitoring, plasmonics, cancer diagnosis, cell imaging, and functional device [17–25]. In this review, we will focus on the application of M13 phage as bio-optical sensor. Initially, we will explain the structural and functional characteristics of M13 phage. Next, the recent progress in optical sensing application of M13 phage, such as immunofluorescence assay, surface plasmon resonance (SPR), Förster resonance energy transfer (FRET), and surface enhanced Raman scattering (SERS) will be introduced. The simple physical and/or optical concept of the corresponding sensing application will also be outlined.

## M13 phage for the immunofluorescence assay application

**Fig. 1:**
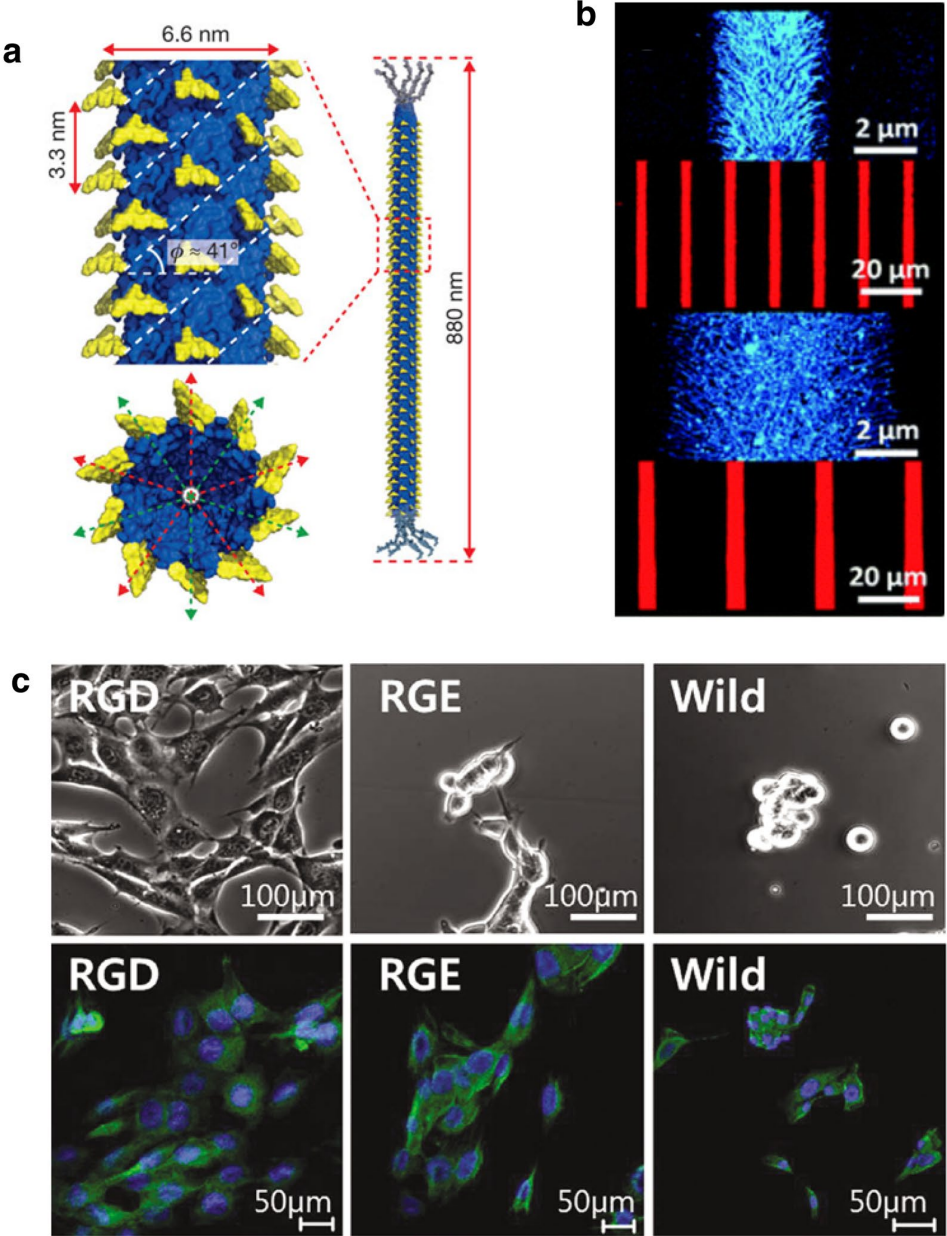
**a** Schematic for structure of M13 phage [32]. Reprinted with permission from Macmillan Publishers Limited and part of Springer Nature © 2016. **b** Fluorescent image of phage proteins [35]. Reprinted with permission from Royal Society of Chemistry © 2016. **c** Fluorescent labelling of various genetic engineered M13 phage (RGD, RGE, and Wild) [36]. Reproduced with permission from American Chemical Society © 2016

As mentioned above, M13 phage has often been used to manage various functional nanomaterials [18, 19, 24, 26–30]. In particular, since the shape of M13 phage is well-defined and can be modified genetically and chemically through the phage display technique to reveal functional peptides [31], M13 phage is particularly useful for various optical applications. Structurally, M13 phage has a cylindrical shape, 880 nm in length and 6.6 nm in diameter (see Fig. 1a) [32]. The single stranded DNA is covered with cylindrical coat made up of 2700 copies of the major coat protein (pVIII), and there are five copies each of the minor protein (pIII and pVI) and other minor proteins (pVII and pIX) at both ends of the cylinder, respectively [33]. Physically, pVIII protein has a helical structure and the shape of phage body is the fivefold rotational and twofold screw symmetry [31]. This precisely defined structure can provide regular molecular spacing. According to recent results, the average distances between two neighboring N-termini of pVIII proteins are 3.2 and 2.4 nm [34].

**Fig. 2:**
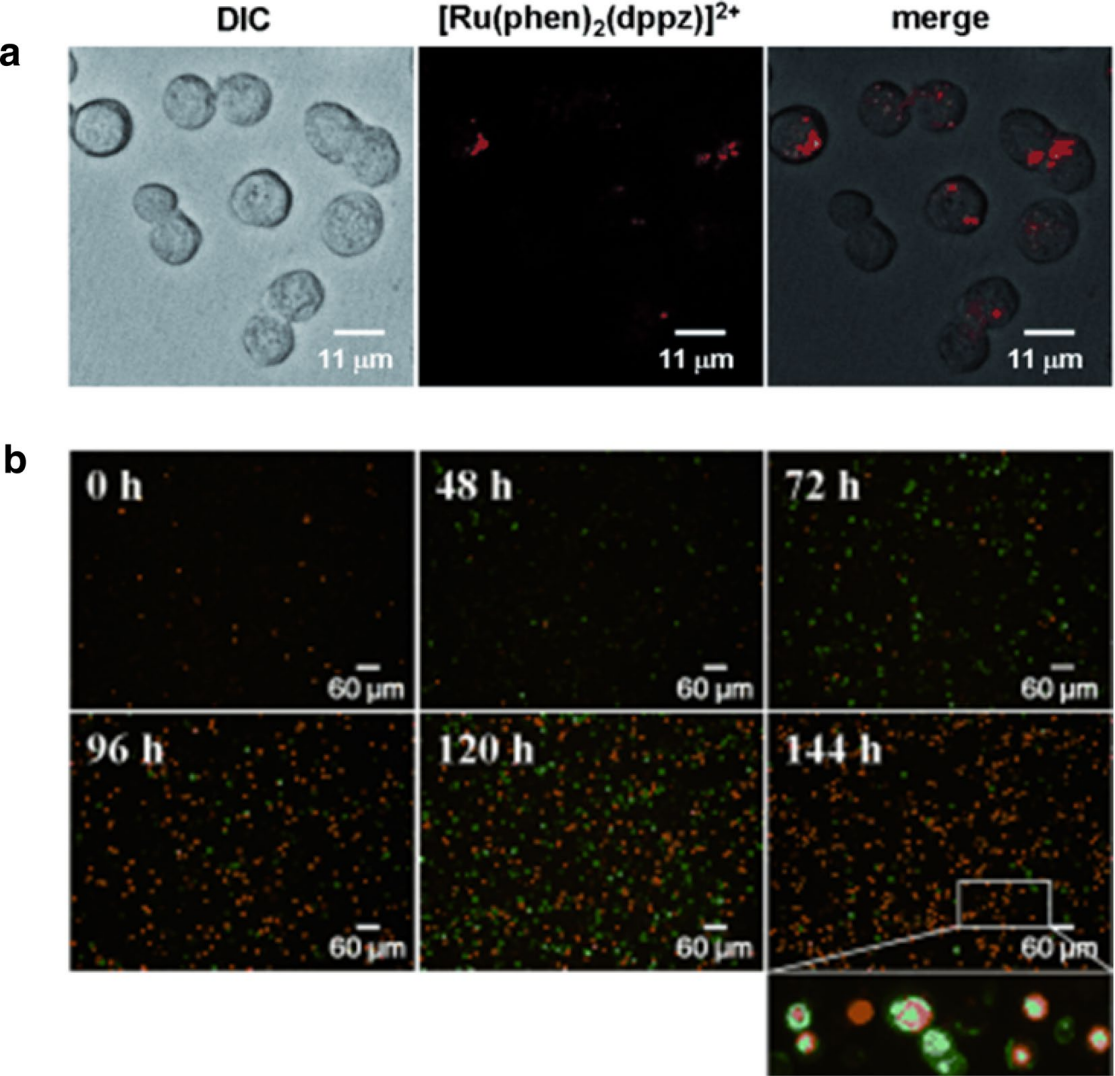
**a** Fluorescent labelling of phage through conjugation with ruthenium complexes. **b** Various time-lapse confocal microscopy images of phage conjugated fluorescent materals. *Red* [Ru(phen)2(dppz)]2+ , *green* GFP, *yellow* colocalization of *red* and *green* fluorescence [37]. Reprinted with permission from Wiley–VCH Verlag GmbH & Co. KGaA, Weinheim © 2016

**Fig. 3:**
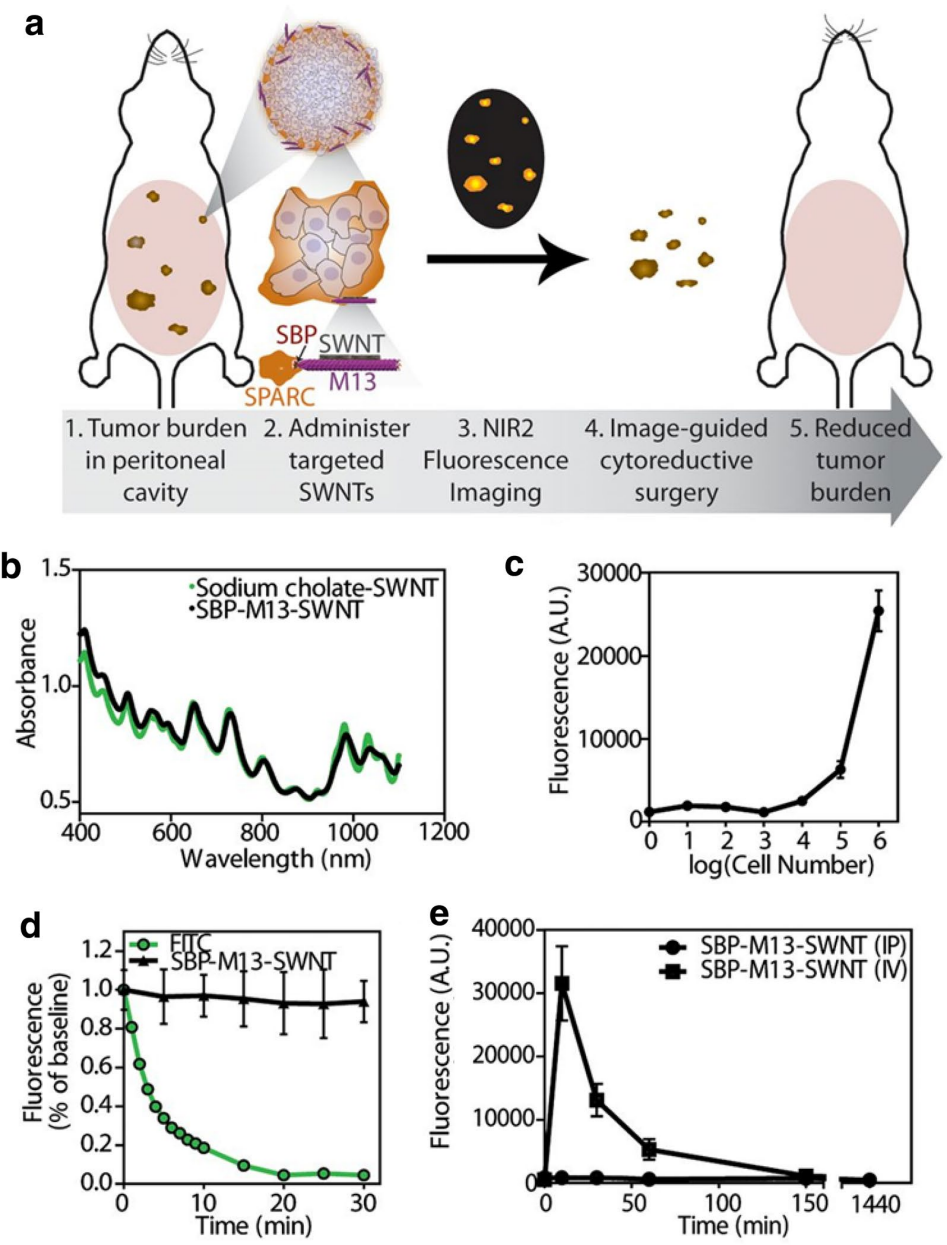
**a** Schematic diagram for tumor-targeting of SBP–M13–SWNT probe. **b** Absorbance spectrum of SWNTs in sodium cholate and SBP–M13–SWNT probe, respectively. **c** Fluorescence intensity of SBP–M13–SWNT in ovarian cancer cell culture. **d** Photobleaching fluorescence decay of FITC and SBP–M13–SWNTs, respectively. **e** Pharmacokinetic circulation study of SBP–M13–SWNT [38]. Reprinted with permission from National Academy of Sciences © 2016

M13 phage can be used as a platform for functional materials (see Fig. 1b, c) [35]. It is in fact a nanofiber whose surface chemistry can be site- specifically modified [1]. By genetic engineering, a peptide or protein of M13 phage can be site-specifically displayed at the tip and/or along the body of the phage. This DNA engineering can leads to the single, double, or triple display with the combination of different peptides or proteins on a single phage [1]. The genetically engineered phage can be used as novel phage-based array chips that are optically readable for cell proliferation and morphology assays. In practice, Yoo et al. have implemented phage-chip arrays using M13 phage that was engineered to display integrin-binding peptide (RGD) on its major coat proteins and/or immobilize FGFb on its minor coat proteins [36]. The authors also monitored cellular proliferation using this phage-chips array with engineered phage to display integrin-binding peptide RGD, a control RGE peptide, or no peptide (wild-type phage). In addition, the fluorescent labelling by conjugation between peptides and different nanomaterials, such as semiconductor nanocrystals and metal complexes, is possible (see Fig. 2a, b) [37]. This labelling of the phage envelope by assembling a characteristic protein with fluorescent material is particularly useful in chemical or biological applications. In addition, the labelling can overcome the photo-bleaching and self-quenching effects suppressing the fluorescence intensity of fluorophores [37]. Recently, Ghosh et al. conducted the imaging using M13 phage in the detection of small, deep tumors for early diagnosis and surgical interventions (see Fig. 3) [38]. Through vivo fluorescence imaging, the authors have detected tumors such as ovarian cancer in mouse. For this implementation, Ghosh and co-authors have synthesized M13-stabilized SWNT (SBP (SPARC binding peptide)–M13–SWNT). Their SBP–M13–SWNT selectively detects secreted protein, acidic and rich in cysteines (SPARC)-expressing tumor nodules in ovarian cancer. In particular, as compared to fluorescein isothiocyanate (FITC), no noticeable loss of fluorescence of SBP–M13–SWNTs by photo-bleaching was observed during this period.

## M13 bacteriophage based SPR sensor applications

Recently, the plasmonic effect in a metallic surface has been widely studied as the method to enhance the optical signal. Surface plasmonic effect is known to enhance optical processes, such as the change in the reflective index of a molecule in a metal surface [39]. The origin of this phenomenon is attributed to the oscillation of the free conduction electrons, that is, surface plasmon (SP), induced by the interaction with the external electromagnetic field [40]. This unique phenomenon provides information for the fundamental interaction, such as the molecular binding between an antibody and a receptor.

SPR is a surface field by the charge density oscillation in a metallic surface, where the free electrons of a conductor are responded by oscillating in resonance with the external electromagnetic wave [40]. In the dielectric/metal interface, the wave equation leads to a dispersion relation for SP which depends on the dielectric constant. Solving the Maxwell equations with a frequency dependent dielectric constant of metal and a dielectric material, the dispersion relation for SP is given by Eq. (1):

1 $$k_{\text{sp}} = \frac{\omega }{c}\sqrt {\frac{{\varepsilon_{\text{d}} \varepsilon_{\text{m}} \left( \omega \right)}}{{\varepsilon_{\text{d}} + \varepsilon_{\text{m}} \left( \omega \right)}}}$$

where *k*_sp_ is the wave vector, *ω* is the optical frequency, *c* is the speed of light, *ε*_d_ and *ε*_m_ are the dielectric constants of dielectric material and metal, respectively [41, 42]. Since metals usually have negative dielectric constants in the UV/VIS range, this equation represents that the momentum of the SP mode is greater than that of a free-space photon at the same frequency: dielectric materials have the positive dielectric constant [43]. This charge density wave is known as the surface plasmon polariton (SPP), which is a non-radiative electromagnetic wave that propagates in the direction parallel to dielectric material interface and a transverse magnetic (TM)-polarized that magnetic field vector is perpendicular to the direction of propagation. Since the SPR frequency is sensitive to the values of the dielectric constant of a dielectric material [44], the SP oscillation is very sensitive to any change in this interface. For example, the resonant coupling with SP and electromagnetic field gives rise to the noble phenomena, such as the enhancement of molecular absorption.

**Fig. 4:**
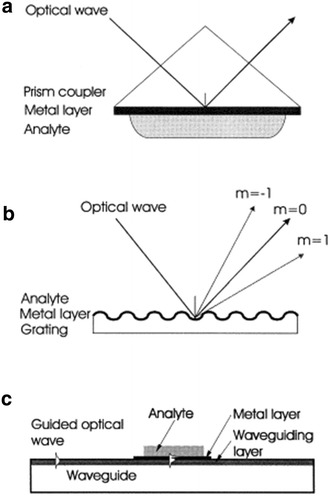
Commonly used configurations of SPR sensors: **a** prism coupler-based SPR system (the Kretschmann configuration), **b** grating coupler-based SPR system, **c** optical waveguide-based SPR system [45]. Reprinted with permission from Elsevier B.V © 2016

The commonly used SPR setups are shown in Fig. 4 [45]. In the Kretschmann configuration, the external electromagnetic field (optical wave) is generally incident through the prism coupler above the metal layer. Then, the photons induce an evanescent wave into the metal layer. While no transport of photons normally occurs through this wave, photons incident at a certain angle are able to pass through the field and excite surface plasmons (SPs) on the opposite side of the metal layer. Whenever analyte absorbs photon, a dip appears in the spectrum of the reflected light at that specific angle. This angle depends on the refractive index of the analyte and, is measured by a spectrometer [46].

In biosensor application, SPR characterizes ultrathin organic and biopolymer films at metal interfaces in a spectrally resolved manner by using its high surface sensitivity. SPR is particularly outstanding in biosensor applications, since SPR can measure the interaction of unlabeled molecules with surface-bound species in real time [47]. In addition, it has advantages of the high specificity and affinity of antibodies to directly detect analytes without additional treatments, such as sample purification or enrichment, competitive immunoassay set-ups, or the use of labeled reagents [48]. Recently, the use of the SPR imaging has been successfully demonstrated in biosensor applications of antigen–antibody reaction, annealing complementary pairs of oligonucleotides, and regulating biological function of DNA interactions [47, 49–57].

**Fig. 5:**
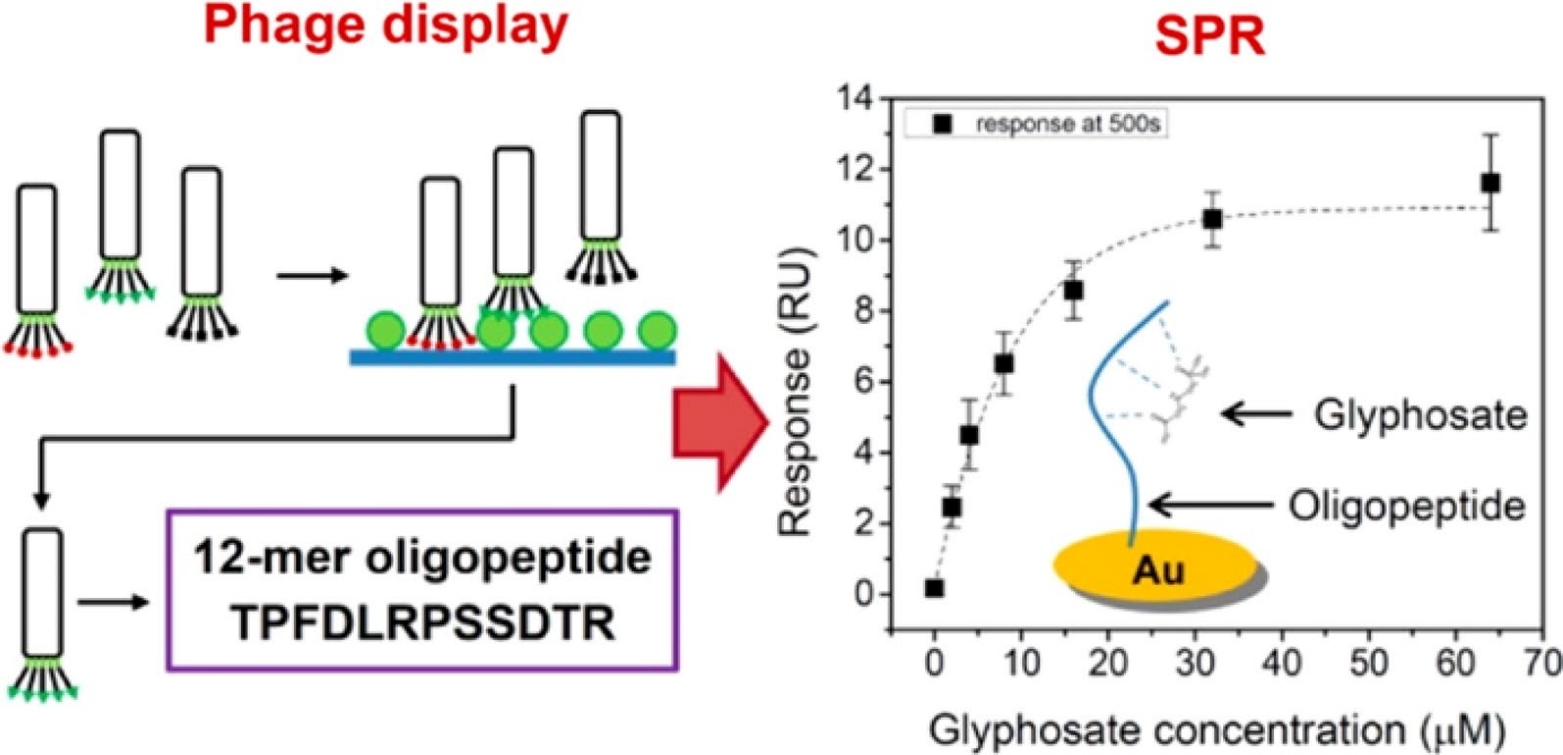
Glyphosate-binding oligopeptide (TPFDLRPSSDTR) and glyphosate concentration dependence of SPR responses [60]. Reproduced with permission from American Chemical Society © 2016

M13 phage is also used as an antibody for various SPR applications. Since the production of antibody allowing the specific detection of a target material is time-consuming and expensive [58], M13 phage, which enables a specific recognition of target, can be used as an alternative. Recently, Karoonuthaisiri et al. reported the SPR assay based on M13 phage [58]. For this application, M13 phage expressing 12-mer peptides was employed as a Salmonella-specific bacteriophage to detect the foodborne bacterium Salmonella. This Salmonella-specific phage-based SPR assay has a very low cross reactivity with other non-target foodborne pathogens and detection limits of 8.0 × 10^7^ and 1.3 × 10^7^ CFU/mL for one-time and five-time immobilized sensors, respectively [58]. The phage-based SPR technique can be used to detect glyphosate (see Fig. 5). Glyphosate (N-(phosphonomethyl)glycine) is a herbicide that is used to remove weeds in farms, parks, and gardens [59, 60]. However, glyphosate as a potential endocrine disruptor can cause several environmental problems [60–63]. Ding et al. reported a SPR biosensor enabling the detection of glyphosate in real time. The oligopeptide, which is prepared by phage display and has a sequence of TPFDLRPSSDTR, is used as a sensing element. It shows the high binding specificity for glyphosate (*K*_D_ = 8.6 μM). For the SPR measurement, modified oligopeptide (TPFDLRPSSDTRGGGC) is immobilized on the gold sensor chip. In the detection of glyphosate, the oligopeptide-based SPR biosensor shows the sensitivity of 1.02 RU/μM and has the limit of detection of 0.58 μM. This SPR biosensor is also compatible to other analytes, such as glycine, thiacloprid, and imidacloprid [60].

These noble applications are attributed to the chemical and biological properties of phages suitable for a real-time sensor development. Therefore, M13 bacteriophage can be used as an attractive platform for the SPR sensing application.

**Fig. 6:**
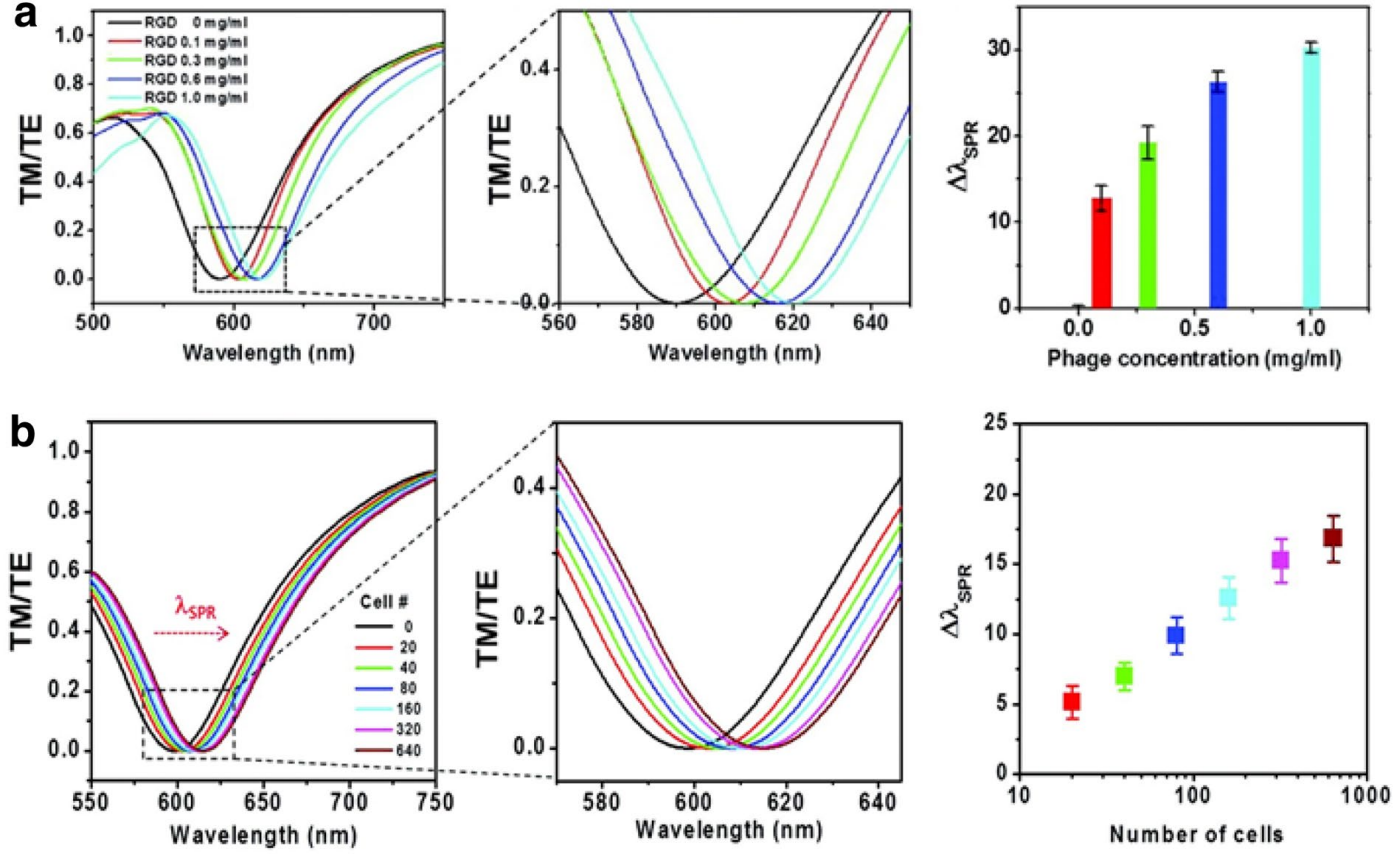
**a** M13 phage (RGD) concentration and **b** Number of NIH3T3 mouse fibroblast cells dependent SPR spectral shift [36]. Reproduced with permission from American Chemical Society © 2016

While previous M13 phage-based studies of the SPR measurement focused on the specific binding property of the functionalized M13 phage, some groups integrated self-assembled property of M13 phage to SPR-sensing applications. Recently, Yoo et al. reported phage-arrays composed of self-assembled structures [36]. To make a uniform phage film, the authors used the pulling up method enabling for the ordered patterning due to the evaporation- induced spontaneous reorganization of phages. In their contribution, phages modified to display peptides of RGD, RGE and HPQ successfully detected NIH3T3 mouse fibroblast cells through a spectral shift in the SPR spectrum [36]. Their phage-chip array shows the phage concentration- and cell numbers-dependent SPR shift. As phage concentration increases from 0.3 to 1 mg/mL, the SPR spectrum is red-shifted due to the increase in sample thickness. Similarly, the increase in numbers of cells influences the spectral shift to a longer wavelength (see Fig. 6). In addition, the authors observed that controlling of cellular direction and morphology by self-assembled monolayer is effective in guiding cell growth [36].

**Fig. 7:**
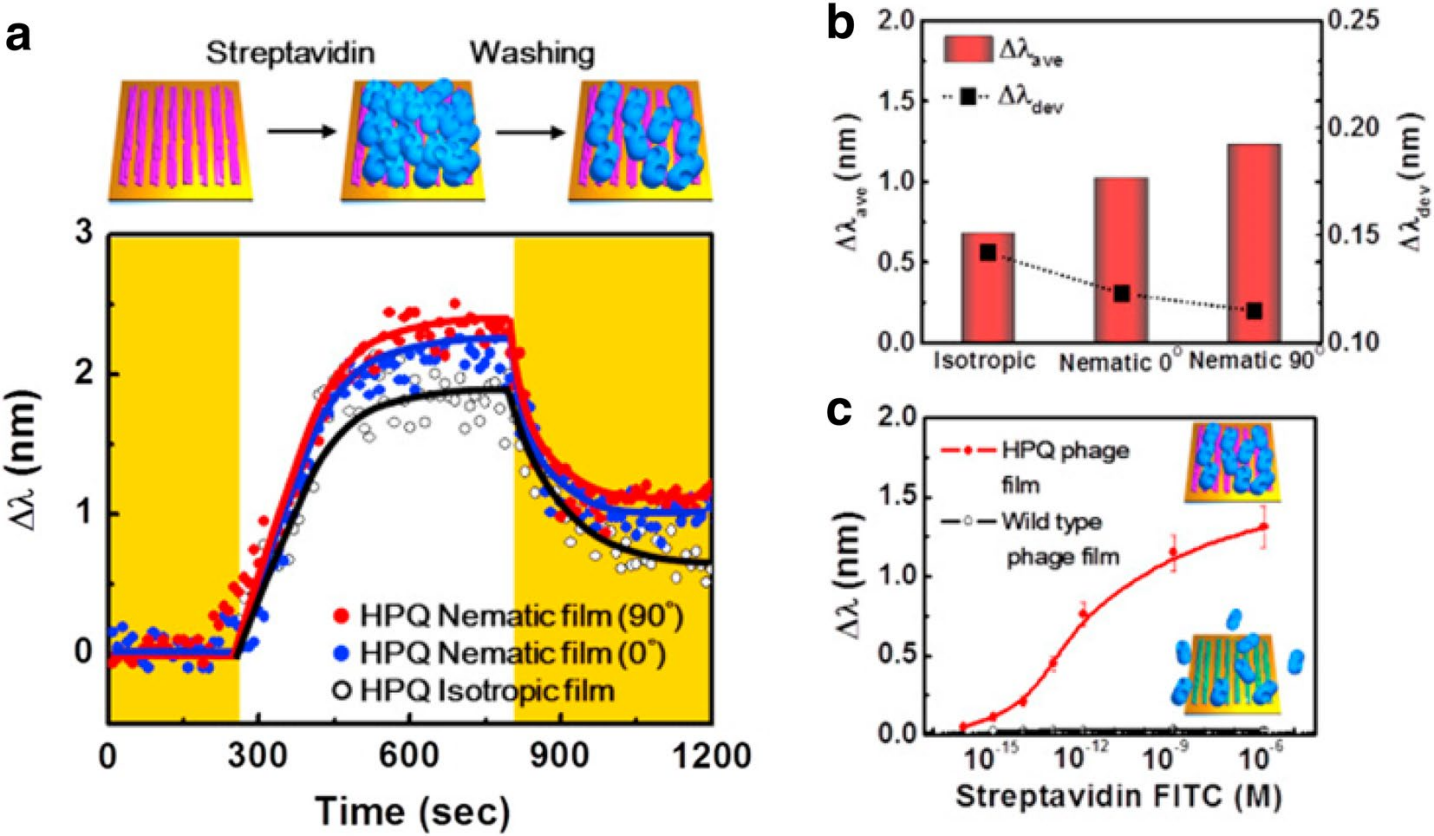
**a** The spectral shift of resonance peak for different types of phage films. **b** The SPR sensitivity comparison between phage films. **c** The selectivity of HPQ phage film for streptavidin FITC [64]. Reprinted with permission from Elsevier B.V. © 2016

Oh et al. have applied the self-assembled property of M13 phage on the SPR sensing application [64]. The authors reported M13 phage based on the novel SPR sensing system including high selectivity for streptavidin. As a sensing material, M13 phage incorporated with specific binding peptide (His-Pro-Gln: HPQ) has been prepared through the phage display technique. The nematic M13 phage matrices have been fabricated on the gold films with the thickness of ~50 nm deposited on glass substrates by a simple pulling technique, which is commonly used for the self-assembly process of liquid particles. Through this fabrication process, Oh et al. have implemented an anisotropic nanostructure by mimicking the 3D photonic crystal structure of Morpho didius. Their system has demonstrated excellent selectivity and sensitivity in the SPR signal by ~2700 copies of genetically expressed peptide on the pVIII major coat protein [64]. Their system has also exhibited the sensitivity dependence for the alignment of receptor matrix in the specific direction. As shown in Fig. 7, different spectral shift of resonance peak is observed for three types of M13 phage films (isotropic, nematic horizontally (nematic 0°), and nematic perpendicularly oriented (nematic 90°) phage films). Since the confinement of the near field differs depending on the orientation due to the anisotropic nature of the self-assembled M13 phage, these results imply that the detecting efficiency of the phage based on the SPR signal can be maximized by analyte concentrations in real time [64]. These applications are attributed to the chemical and biological properties of M13 phage suitable for a real-time sensor development. Therefore, M13 phage can be used as an attractive platform for the SPR sensing application.

## M13 phage-based FRET applications

FRET has been most widely investigated in various applications of fluorescence, including medical diagnostics, DNA analysis, and bio-optical imaging [65]. After this phenomenon, named by Theodor Förster, was initially described in 1948 [66], FRET-based studies have expanded into other research areas with the help of advances in the fluorescence detection technique by the improved spectral resolution and high sensitivity. A typical aspect of these applications involves the selection of probe materials suitable for the optimization of the energy transfer. For this reason, many studies and developments associated with fluorescent materials applicable to FRET, such as organic dyes, conjugated polymers, semiconductor nanocrystals, and quantum dots (QDs), have been performed. For example, due to their electron affinity and high quantum efficiency, organics dyes are most commonly used as efficient fluorescent materials in FRET based on optical detection. For decades, it has been proven that organic dyes offer several unique advantages in FRET-based biomolecular imaging application [67–71]. Furthermore, due to their unique electrical and optical properties, conjugated polymers have also received more attention as probe materials in the investigation of the FRET mechanism. Conjugated polymers have a unique structure characterized by a π-orbital enabling exciton hopping along their backbone [65, 72–82]. In addition to the use of organic molecules, recent research has suggested that colloidal semiconductor nanocrystals or QDs are also useful for FRET applications [83–91], because they have many advantages as compared to conventional organic fluorophores, such as high extinction coefficient and size tenability [9, 14, 92–96].

Although the fluorescent materials for FRET have noticeable advantages, such as high quantum efficiency and electron affinity, the optical properties of these materials do not guarantee the optimal energy transfer, because the carrier relaxation is affected by the quenching process which diminishes fluorescence intensity or by trapping excited carriers. This quenching process is originally caused by molecular contact, and the common molecular system generally has many quenchers. Thus, the appropriate work of the system design is very important for efficient FRET. Recently, in situ FRET based on the optical DNA detection scheme using conjugated polymer has been demonstrated by Bazan and many other researchers [97–102]. They suggested the excellent FRET design enabling the amplification of fluorescence signal through the fine tuning of the intermolecular distance by the electrostatic interaction between optical platforms. Thus, their result also implies that the optimal FRET is possible through a careful system design.

In M13 phage based on sensor applications, M13 phage is commonly used as an alternatives to classical 1D nanoscaffolds, such as carbon nanotubes, while providing suitable constructs serving as heterogeneous supports of nanoparticles (NPs), a high surface area template for the co-anchoring of photo-activated molecular donors/acceptors, a spacer element to funnel and direct the sequential electron-transfers [103]. In particular, due to its due specific binding property and well-defined shape, M13 phage is useful as a platform or scaffold. Thus, we can easily expect that M13 phages can be used as optical platforms for FRET.

In this section, we will introduce the use and potential of M13 phage in FRET-based optical sensor application. For this purpose, we will first explain the basic equations of FRET for understanding the essential concept of FRET. Then, we will account for the recent progress in FRET application based on M13 phage.

**Fig. 8:**
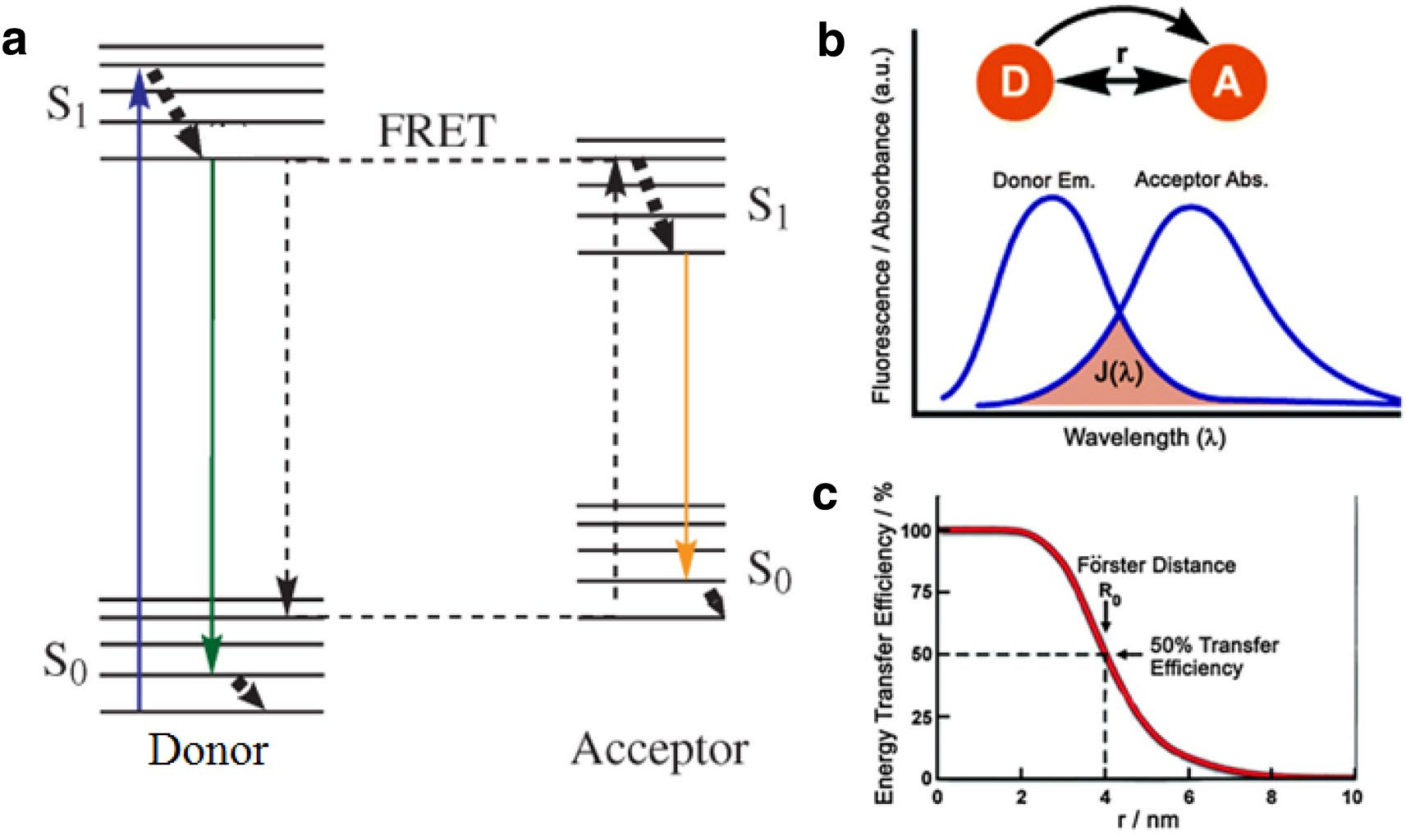
**a** Jablonski diagram of FRET process [104]. Reproduced with permission from MDPI AG © 2016, **b** The schematics for distance (r) dependence of FRET and spectral overlap. **c** Förster distance and FRET efficiency [105]. Reprinted with permission from Royal Society of Chemistry © 2016

Theoretically, FRET is the excitation energy transfer process from the excited donor molecule to an acceptor molecule by the dipole–dipole coupling and it is observable at the range of 10–100 Å as shown in the Jablonski diagram (see Fig. 8a) [104]. When a donor molecule is excited by incident light, the excited state energy of donor can be transferred to an acceptor molecule which is in close proximity. Then, this leads to a decrease in the donor’s fluorescence intensity and an increase in the acceptor’s emission intensity. Interestingly, the resonance energy transfer process non-radiatively occurs without the involvement of a photon, although the emission spectrum of donor molecules overlaps with the acceptor’s absorption spectrum. In addition, the energy transfer is a through-space interaction which is mostly independent from the intervening solvent and/or macromolecule.

In FRET, the energy transfer process is based on the concept of coupled dipoles, which can exchange energy with another dipole with a similar resonance frequency [65]. Thus, the strength is determined by the relative orientation and distance between two dipoles. Assuming that two molecules (donor and acceptor) are separated by a distance *R*, the FRET rate is inversely proportional to sixth power of *R*. The rate equation is given by Eq. (2) [65].

2 $$k_{\text{FRET}} (R) = \frac{{Q_{\text{D}} \kappa^{2} }}{{\tau_{\text{D}} R^{6} }}\left( {\frac{{9000\ln \left( {10} \right)}}{{128\pi^{5} N_{\text{A}} n^{4} }}} \right)\frac{{\mathop \smallint \nolimits_{0}^{\infty } F_{\text{D}} \left( \lambda \right)\alpha_{\text{A}} (\lambda )\lambda^{4} d\lambda }}{{\mathop \smallint \nolimits_{0}^{\infty } F_{\text{D}} \left( \lambda \right)d\lambda }}$$

where *Q*_D_ is the quantum yield of the donor in the absence of acceptor, *τ*_D_ is the decay time of donor’s fluorescence in the absence of acceptor, *κ* ^2^ is a factor considering the relative orientation of two dipoles; *κ* ^2^ = 2/3 considering molecular averaging at liquid solution, *N*_A_ is Avogadro’s number, and *n* is the refractive index of medium. In case of solution, it corresponds to the refractive index of the solvent. *F*_D_(*λ*) is the donor’s fluorescence intensity in the absence of acceptor and *α*_A_(*λ*) is the extinction coefficient of the acceptor at wavelength *λ*. The integral part represents the degree of spectral overlap between the donor’s emission and the acceptor’s absorption. Figure 8b schematically shows the distance dependence of FRET and spectral overlap [105]. This rate equation can be modified by the Förster characteristic distance (*R*_0_) as given by Eq. (3) [65].

3 $$k_{\text{FRET}} (R) = \frac{1}{{\tau_{\text{D}} }}\left( {\frac{{R_{0} }}{R}} \right)^{6}$$

The Förster distance (*R*_0_) of Fig. 8c is the intermolecular distance between two molecules when the energy transfer efficiency is 50 % and this can be simply calculated from the spectral properties of the donor and the acceptor [105]. The distance-dependent nature of FRET is very useful in chemical or biological applications, because this process occurs over distances comparable to the dimensions of biological macromolecules [106]. Therefore, FRET is suitable for quantitatively detecting the conformational change in the orientation of fluorescent molecules and obtaining structural information about the macromolecule. For this reason, FRET is described as “a spectroscopic ruler” to be a proximity indicator [107].

**Fig. 9:**
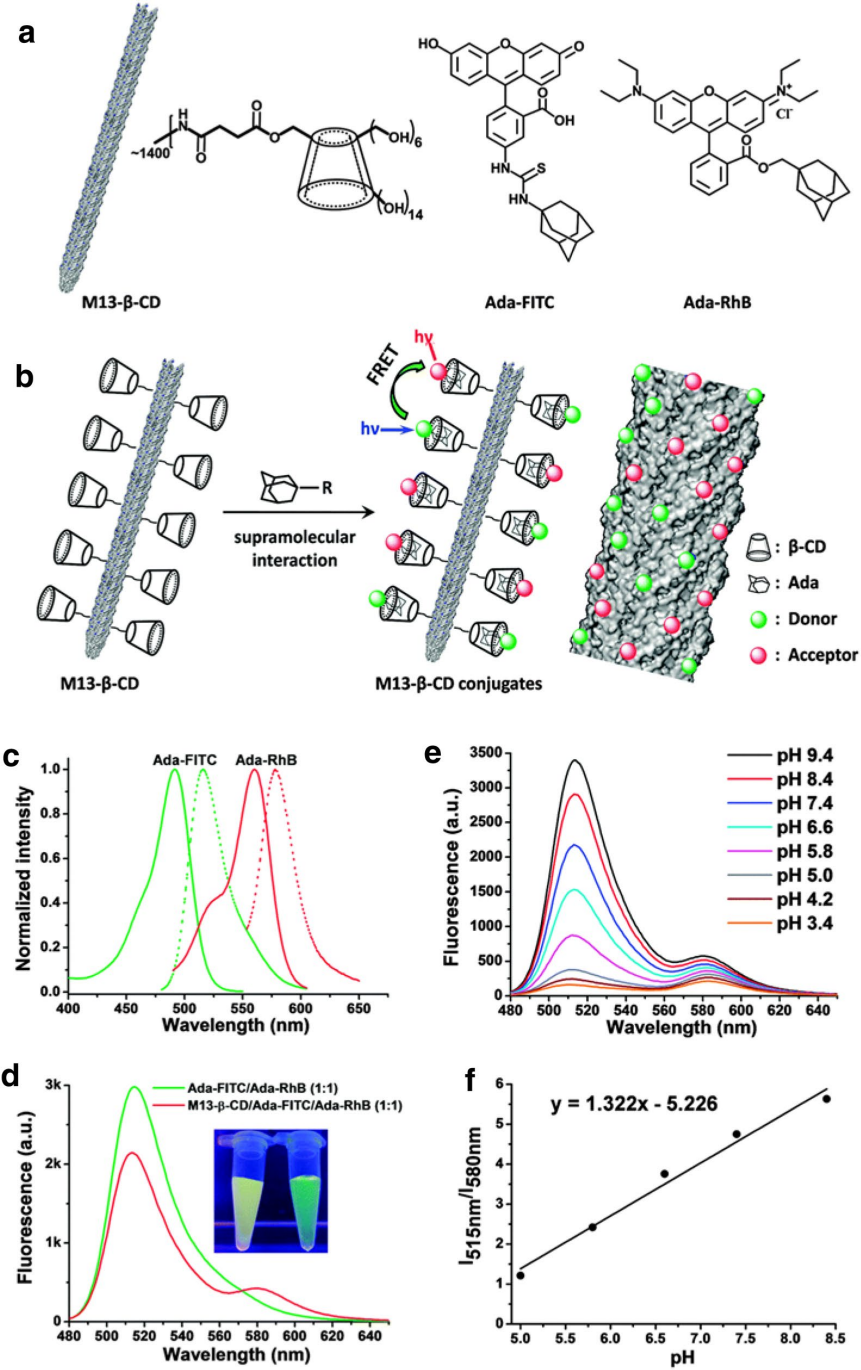
**a** The structures of M13-β-CD, Ada-FITC, and Ada-RhB. **b** Schematic of the FRET based ratiometric fluorescent pH nanosensor. **c** Normalized absorption spectra of Ada-FITC (*green solid*) and Ada-RhB (*red solid*), normalized fluorescence spectra of Ada-FITC (*green dot*) and Ada-RhB (*red dot*), respectively. **d** Fluorescence spectra of the mixture of Ada-FITC and Ada-RhB (*green*) or M13-b-CD/Ada-FITC/Ada-(*red*) under 450 nm excitation. The *inset* shows the image of solution in the absence (*green*) and presence (*yellow*) of M13 phage. **e** pH dependence of M13-β-CD/Ada-FITC/Ada-RhB complex. **f** The peak emission ratio between Ada-FITC (515 nm) and Ada-RhB (580 nm) shows linear pH dependence [34]. Reprinted with permission from Royal Society of Chemistry © 2016

Recently, Chen et al. have reported FRET based on ratiometric fluorescent nanosensors using M13 phage [34]. They have used M13 phage as a scaffold to construct FRET-based ratiometric fluorescent nanoprobes. As a FRET donor and an acceptor, fluorescein isothiocyanate (FITC) and rhodamine B (RhB) are used, respectively. Fluorescent dyes are conjugated to the N-terminus at the exterior surface of M13 phage using β-Cyclodextrin (β-CD) as a molecular linker (see Fig. 9). In general, there are the proper spectral overlaps between FITC and RhB, where the FRET process from FITC to RhB would occur. Thus, the changes in the fluorescence spectra by FRET can be expected, if intermolecular distance between dyes is sufficiently close for FRET. In the presence of M13-β-CD, the significant increase in the emission intensity of RhB (580 nm) was observed [34]. Considering that the average distances between two neighboring N-termini of pVIII proteins are 3.2 and 2.4 nm [34], this result clearly indicates that peptides of M13 phage provide a proper molecular spacing for efficient FRET. In addition, Chen and co-authors have implemented M13 phage-based ratiometric sensor using the sensitivity of dyes for acidity: FITC is pH sensitive, while RhB is not. Therefore, M13 phage is a suitable optical platform for the FRET-based applications.

**Fig. 10:**
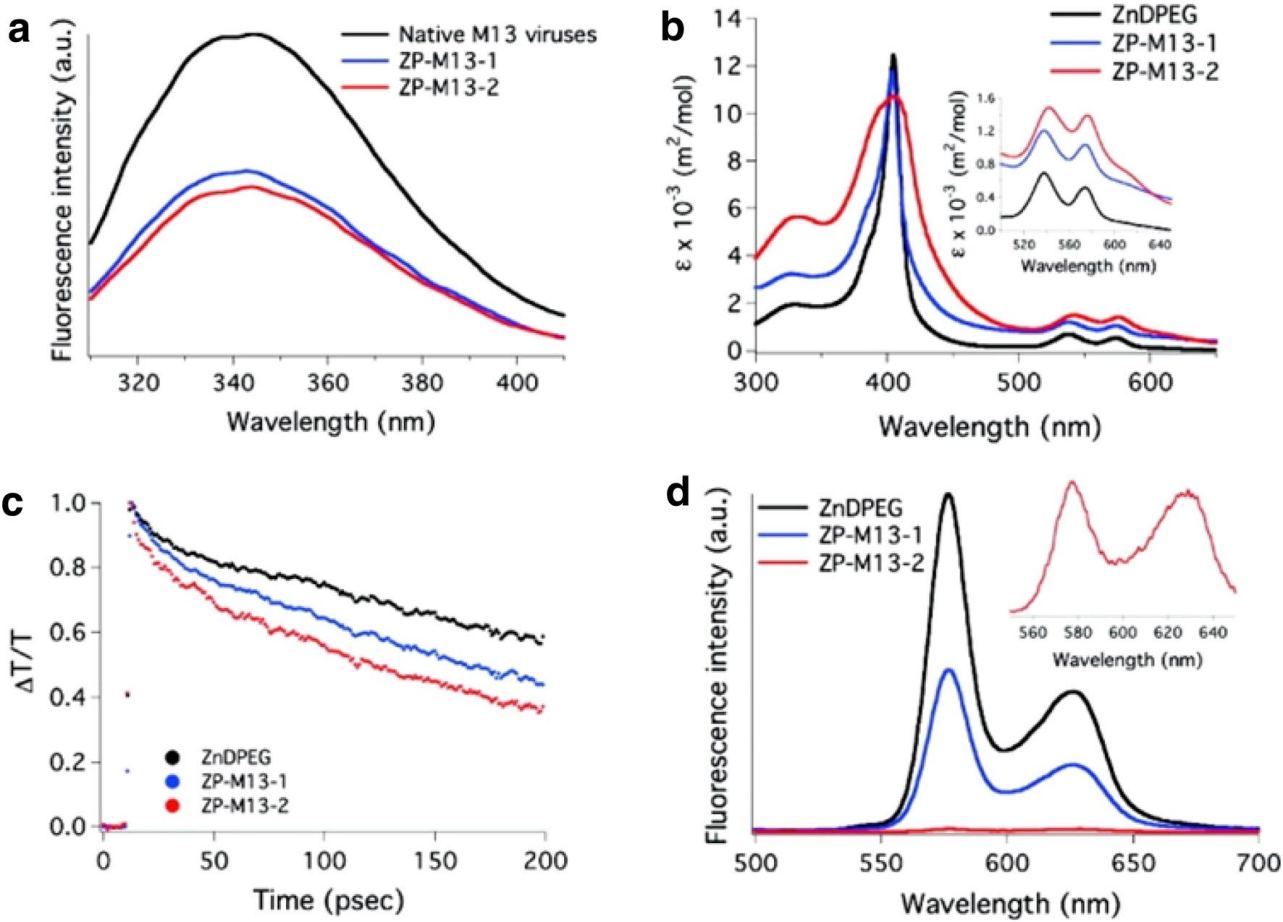
**a** Tryptophan fluorescence emission spectra of native M13 phage and ZP-M13 under 295 nm excitation. **b** Molar extinction coefficient (absorption), **c** transient absorption at 400 nm, and **d** fluorescence spectra of ZnDPEG, ZP-M13-1, and ZP-M13-2 under 400 nm excitation. The *insert* in **b** shows a magnified emission spectrum around 550 nm, and the *insert* in **d** shows a magnified emission spectra of ZP-M13-2 [108]. Reproduced with permission from American Chemical Society © 2016

As other optical platform application of M13 bacteriophage, it can be used as a template for light harvesting or exciton transporting. Recently, Nam et al. have demonstrated a light-harvesting antenna system using M13 phage [108]. Since further modifications and genetic engineering over phage was pivotal in terms of tuning the assembly geometry and chromophore distances [103], the authors have noticed that the ordered coat protein of M13 phage can serve as a template guiding the interaction between pigments. The authors used Zn (II) deuteroporphyrin IX 2,4-bis (ethylene glycol) (ZnDPEG) as a model pigment and synthesized two samples, ZP-M13-1 (1564 porphyrins) and ZP-M13-2 (2900 porphyrins) with different numbers of zinc porphyrins [108]. In this light harvesting system, Nam and co-authors observed the temporal migration of carriers (excitons) along the pigments assembled on the virus using the transient absorption measurement. In the presence of M13 phage, ZP-M13 rapidly decays as compared to ZnDPEG. The lifetime of ZP-M13 is two times shorter than that of ZnDPEG (see Fig. 10). This change results in the delocalization of the excitons driven by FRET, since the modification of the site energy (spectral broadening of absorption spectrum) by intermolecular interaction between protein and porphyrins has clearly influenced the pathway of excitation energy transfer.

**Fig. 11:**
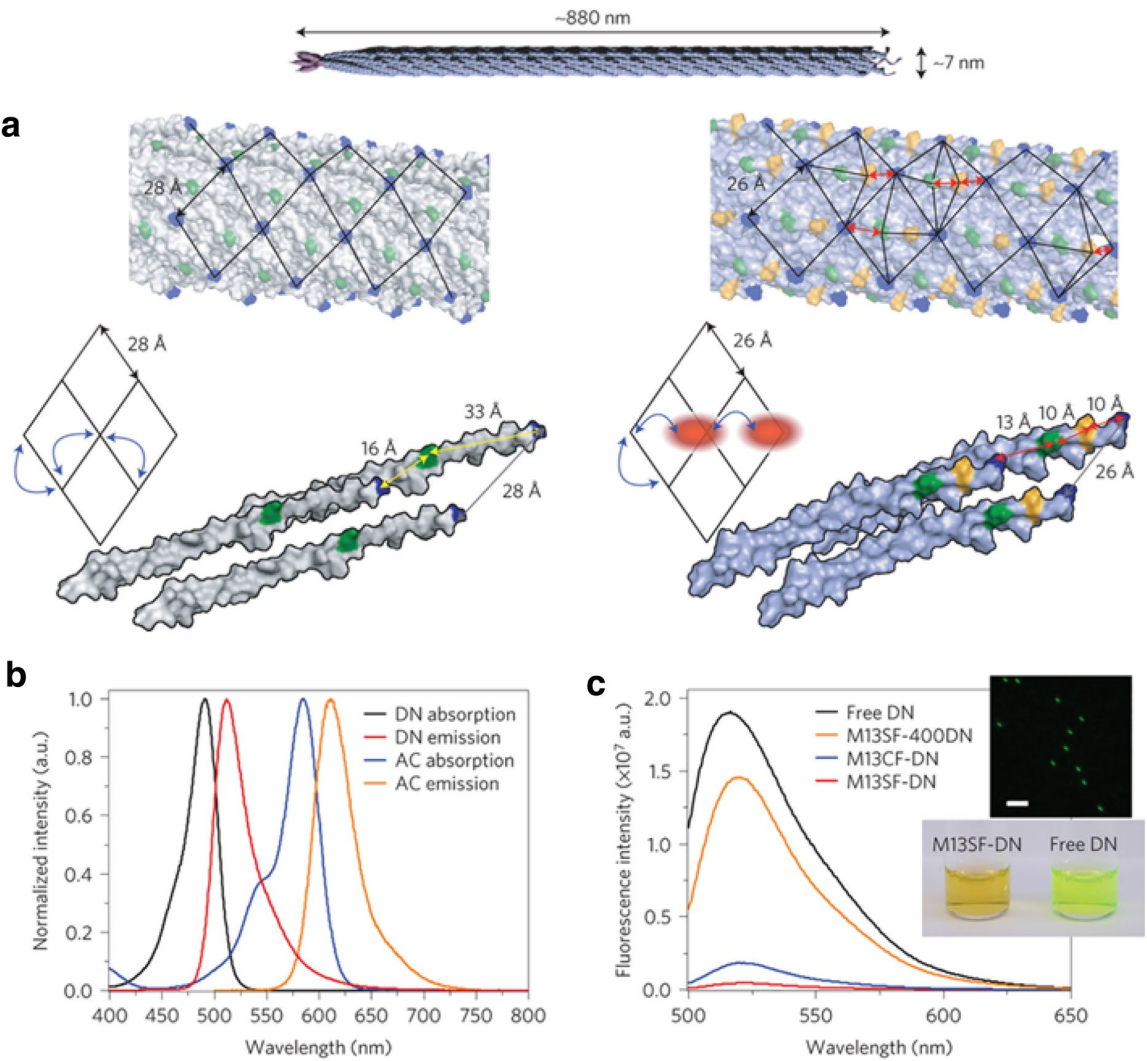
**a** Schematics for exciton transporting mechanism in the genetically engineered M13 phage based light harvesting system. Magnified M13 phage surfaces show the energy-transfer networks between chromophore-binding sites for M13CF (*left*) and M13SF (*right*), respectively. *Insets* show schematic networks of energy transport by exciton hopping in the M13CF and the M13SF, respectively. **b** Absorption and emission spectra of free donor (DN) and free acceptor (AC). **c** Fluorescence spectra of donor under 495 nm. Significant fluorescence quenching is observed in the presence of M13 phage. The *upper inset* is a fluorescence microscope image of the M13SF-DN. The *bottom inset* shows the change in emission colour of donor molecule as FRET occurs [109]. Reprinted with permission from Macmillan Publishers Limited, part of Springer Nature © 2016

Park et al. have also demonstrated the exciton transporting mechanism in M13 phage using organics dyes [109]. They have fabricated a light harvesting system enabling molecular wire effect along to the coat protein of M13 phage. As a scaffold for the fluorophores, M13 phage (M13CF, ADSPHTELPDPAK) engineered by modifying the amino acid sequence of the major coat pVIII protein were used as shown in Fig. 11 [109]. However, unlike in a previous study [108], Park et al. have directly controlled molecular spacing using M13 phage with an additional binding site of 10 Å distance (M13SF, AENKVEDPAK). This binding site contributed to the occupation probability of donor molecules over the possible site per M13 phage. This led to the enhancement of the coupling strength of the bound fluorophores and suppression of the fluorescence quenching via short-range Dexter exchange interaction [109]. In the presence of M13 phage, the fluorescence intensity of Alexa Fluor 488 (donor) is significantly quenched by FRET, while the yellow emission of free donor has no change (see Fig. 11). In addition, due to the strong electronic coupling and the effective energy transport by subsequently highly linked network between the binding sites, the exciton lifetime (~422 ps) of donor is significantly faster than that of free donor (~4 ns). Therefore, the controlling of specific binding sites by inserting or deleting amino acids on pVIII of M13 phage is very useful for FRET based on sensing or higher-level exciton transporting.

## M13 phage-based SERS applications

SERS is a powerful spectroscopic technique enabling for a highly sensitive detection due to the significant amplification of Raman signal from molecules attached to the metallic surface with nanometer size [110, 111]. It has been widely used in various bio- or chemical sensing applications for probing of single molecules [111–113], molecular analysis [114], bio markers [115–119], and environmental monitoring [120, 121]. It has even been used in forensic science for detection of explosives, drugs, blood, DNA, and fingerprints [122], since the Raman spectra of pyridine on silver electrode were first observed in 1974 [123] and their mechanism was analyzed in 1977 [124]. In particular, the SERS field has dramatically progressed by virtue of a high sensitivity with theoretical signal enhancement factor of 10^11^ in an optimized substrate [110].

Before discussing M13 phage-based SERS applications, we first explain the Raman effect and the theoretical mechanism of SERS. The Raman effect is an inelastic scattering process between a photon and a molecule. When light is incident in a molecule, some of the incoming photons are inelastically scattered from a molecule. These scattered photons have a different energy (frequency) as compared to that of incident photons by interaction with molecular vibrational energy states in molecules. This phenomenon appears to frequency-shifted signal (spectral shift to lower or higher energy) in the spectrum. It is called the Stokes scattering when scattered photons lose energy and the reverse process where photons gain the energy due to interacting with excited vibrational states is called anti-Stokes scattering [125]. In comparison to the Raman scattering or Raman, SERS is incorporated with metal nanostructures [126]. In the presence of nanostructures with coinage metals, the excitation of localized surface plasmon resonance (LSPR) can induce the amplification of scattered electromagnetic field [110]. By |*E* ^4^| approximation, the SERS intensity is proportional to the fourth power of incident electric fields (*E*(*ω*_inc_)): *I*_SERS_ ≈ |*E*(*ω*_inc_)|^4^ [126]. In SERS, the enhancement of the Raman signal is explained by two mechanisms of electromagnetic [127] and chemical theories [128, 129]. The electromagnetic approach describes signal enhancement with the amplification in the electric field due to roughness or shape of the metallic surface. In general, an incident electromagnetic plane wave can excite localized surface plasmons confined to the metal surface. Then, the plasmon oscillation perpendicular to the surface can occur, scattering of photons by the resonance with an incident electromagnetic wave. Therefore, SERS is strongly influenced by the systems which can absorb the photon and store the electromagnetic energy into the surface plasmon, i.e., small metal features and gratings [127]. The theoretical electromagnetic enhancement factor is in the order of 10^10^–10^11^ [110]. In the chemical approach, the enhancement of the Raman signal results in the transient charge transfer by the electronic interaction between metal and adsorbate, since a new electronic state by chemisorption can serve as a resonant intermediate state in the Raman scattering [129]. In this case, theoretical chemical enhancement factors are in the order of 10^3^ [110].

Due to the specific binding property of M13 phage, it often used as an analytic material in the SERS applications for biosensing. Recently, Lee et al. reported a biosensor system that integrates SERS-active metal nanoparticles with DNA-modified M13 bacteriophage [130]. In this application, M13 phage is used as a platform for signal enhancement. The authors observed that the high capturing of SERS-active Au@Ag core–shell nanoparticles by single phage leads to the exponential enhancement in the Raman signal [130]. Therefore, they the DNA-phage system shows a 75-fold increase in the Raman signal as compared to that of DNA-antibody due to the high surface area of the phage.

**Fig. 12:**
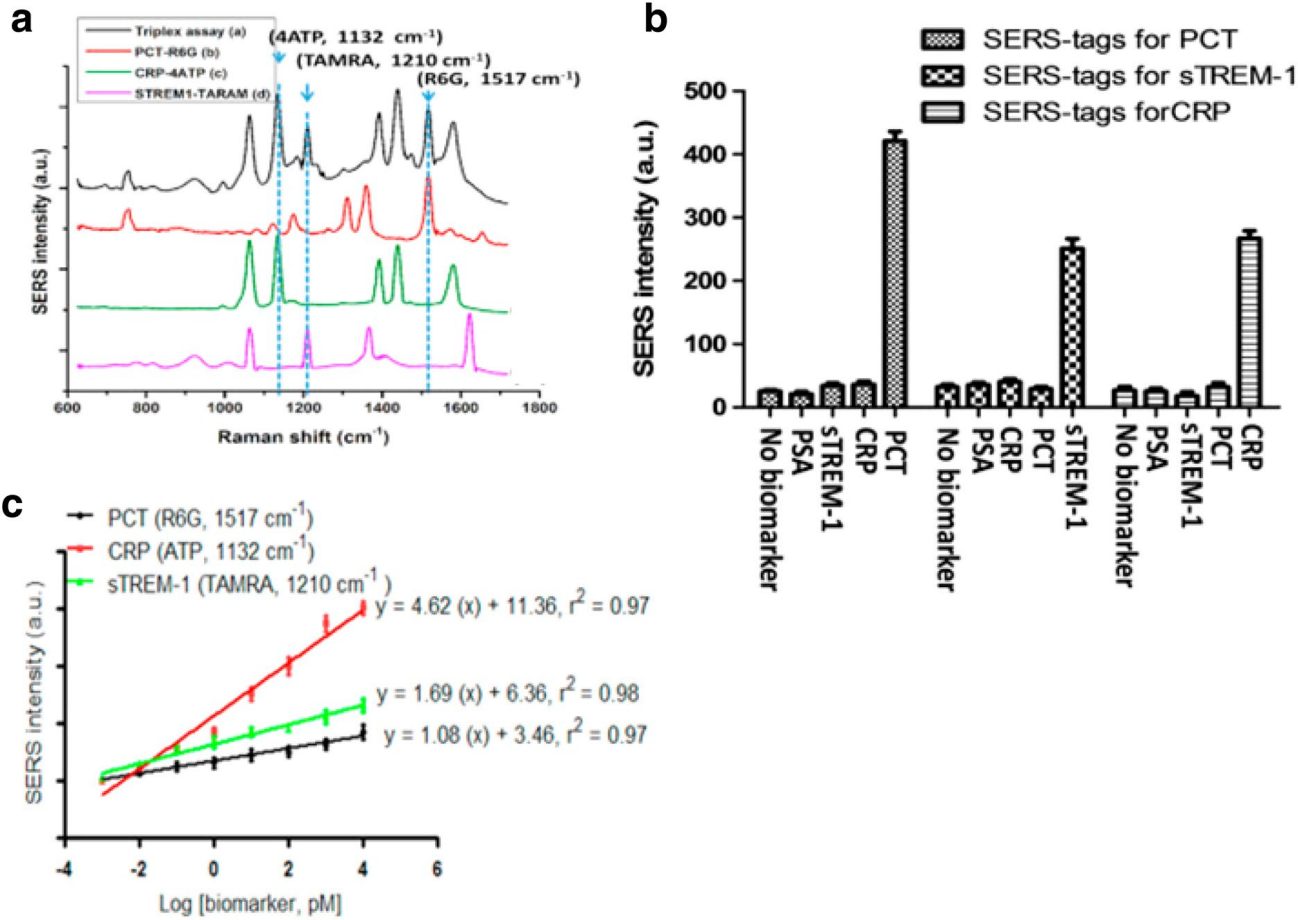
**a** SERS spectra for three sepsis-specific biomarkers (C-reactive protein, procalcitonin, and sTREM-1). **b** Detection of unspecific and cross-talk assay for the biomarker-based sepsis. **c** The detection limit based on concentration of each biomarkers is estimated by Linear fitting of peak intensities. [131]. Reprinted with permission from Elsevier B.V. © 2016

M13 phage can be used as a signal reporter for the SERS-based medical applications; for example, Nguyen et al. reported a new mesoporous SERS substrate using M13 phage modified with cysteine-rich peptides on the pVIII major coat protein for sepsis diagnosis [131]. In their new system, the authors prepared silica mesoporous templates for SERS through polymerization by mixing between M13 phage displaying cysteine-rich peptide and silica precursors. The authors carried out detection for three typical sepsis-specific biomarkers, including C-reactive protein, procalcitonin, and sTREM-1 based on principles of immunoassays (see Fig. 12) [131]. Therefore, they observed that the SERS spectrum shows distinct peaks for each tags and has the detection limit of 27, 103, and 78 pM for each sepsis-specific biomarker. Furthermore, Lentini et al. reported the SERS material based on Phage–Ag nanoparticles for identification of Histiocytic lymphoma cell line (U937) [132]. U937 is an in vitro model cell line for cancer diagnosis in biomedical research. In their contribution, the phage display technique of a 9-mer pVIII M13 phage is used to screen over U937 and silver nanoparticles are incubated with phage clones to acquire the SERS signal. Therefore, the authors found that assembled network between phage-displayed peptides with EIII1 alignment and Ag nanoparticles shows new Raman scattering peaks at 862.6, 1132, and 1154 cm^−1^ as compared to the fundamental feature of U937, as well as signal enhancement [132].

## Summary

In the above-mentioned M13 phage-based optical sensing applications, there are difficulties to directly compare the sensing performance. Specifically, intrinsically different sensing mechanisms and techniques are used, while sensitivity, selectivity, pros and cons of a biosensor are crucial factors. In addition, these techniques were applied to the different sensing ranges, for example, pg/mL to ng/mL level for SPR and ng/mL to μg/mL level for FRET [133]. To provide a more comprehensive picture of prior research, sensing parameters used in previous studies are summarized in Table 1.

**Table 1:** M13 phage-based optical sensors

Sensing technique	Probe	Analyte	Detection limit	Ref.
Immunofluorescence assay	RGD peptide SPARC binding peptide	M13 bacteriophage	– pH 4.5–8.5	[36] [38]
SPR	Salmonellar specific peptides Oligopeptide RGD peptide HPQ peptide	M13 bacteriophage	8.0 × 10^7^ CFU/mL 0.58 μM 0.3 mg/mL 10 fM	[58] [60] [36] [64]
FRET	FITC and RhB Alexa Fluor 488 and Alexa Fluor 594 Zinc porphyrins	M13 bacteriophage	pH 5.0–8.4 – –	[34] [108] [109]
SERS	Cy3 labeled DNA Raman dyes-bound immunogold colloids FITC	M13 bacteriophage	10 nM 27 pM 1 pM	[130] [131] [132]

## Conclusions

In recent years, M13 bacteriophage has expanded its use into various novel research areas, such as fluorescence, SPR, and exciton transporting network. In these sensing applications, versatility of M13 phage is attributed to its nontoxic, well-defined shape, self-assembling, and specific binding properties. Considering the recent trend of the use of M13 phage in bio-optical applications, M13 phage is consequently the most powerful candidates that corresponds to the necessity of the development of sensor enabling detection of various analytes, such as explosives, proteins, DNA, cancers, bacteria, toxins, and metal ions. In addition, since M13 phage is robust, thermally and chemically stable, as well as easy to incorporate with other motifs, such as biomolecules or nanoparticles, via genetic engineering, it is very useful as a functional nanomaterial for more diverse applications. For this reason, M13 phage based on sensor will be consistently developed and lead to new optical sensing strategies for the rapid, accurate, selective, and sensitive detection of analytes incorporated with various spectroscopic methods.
